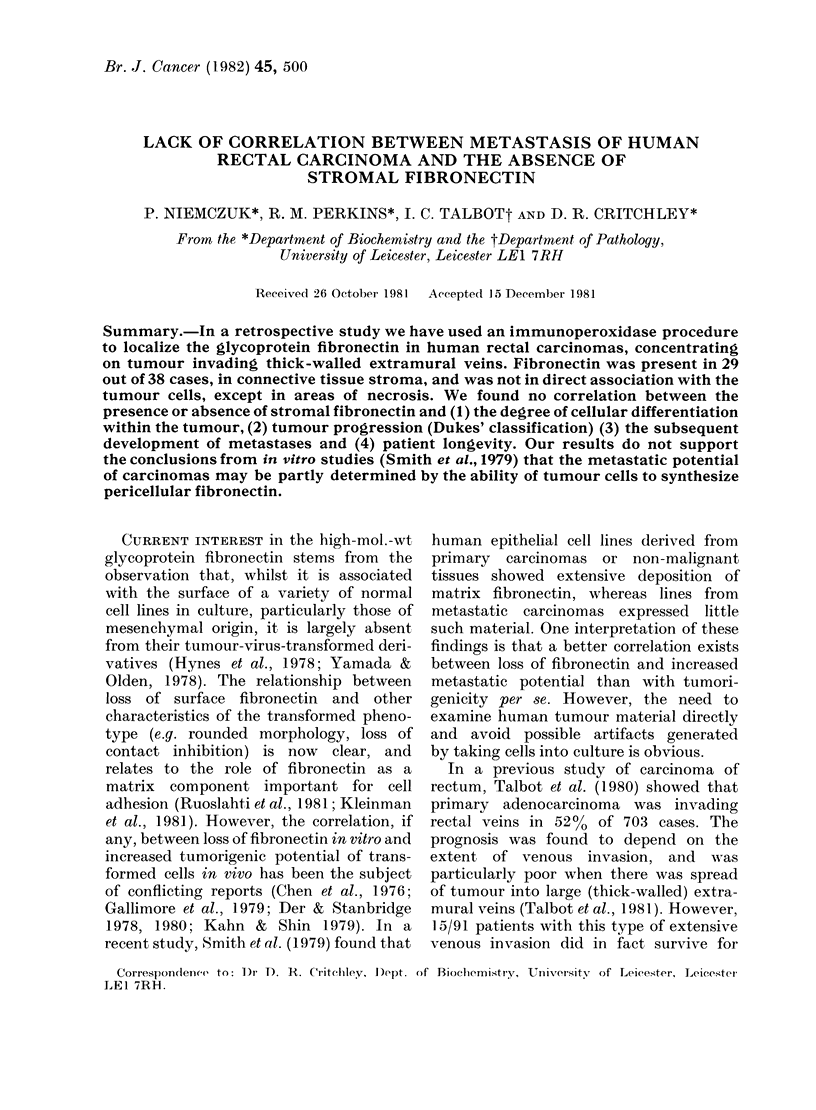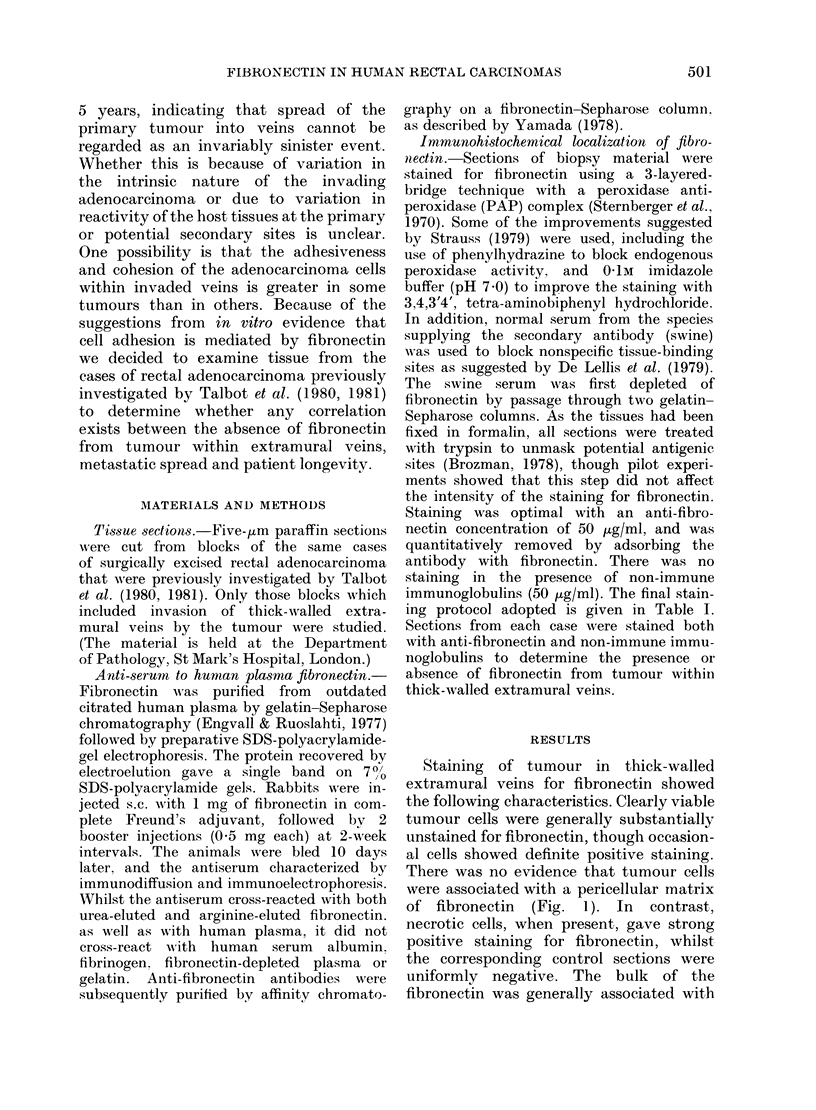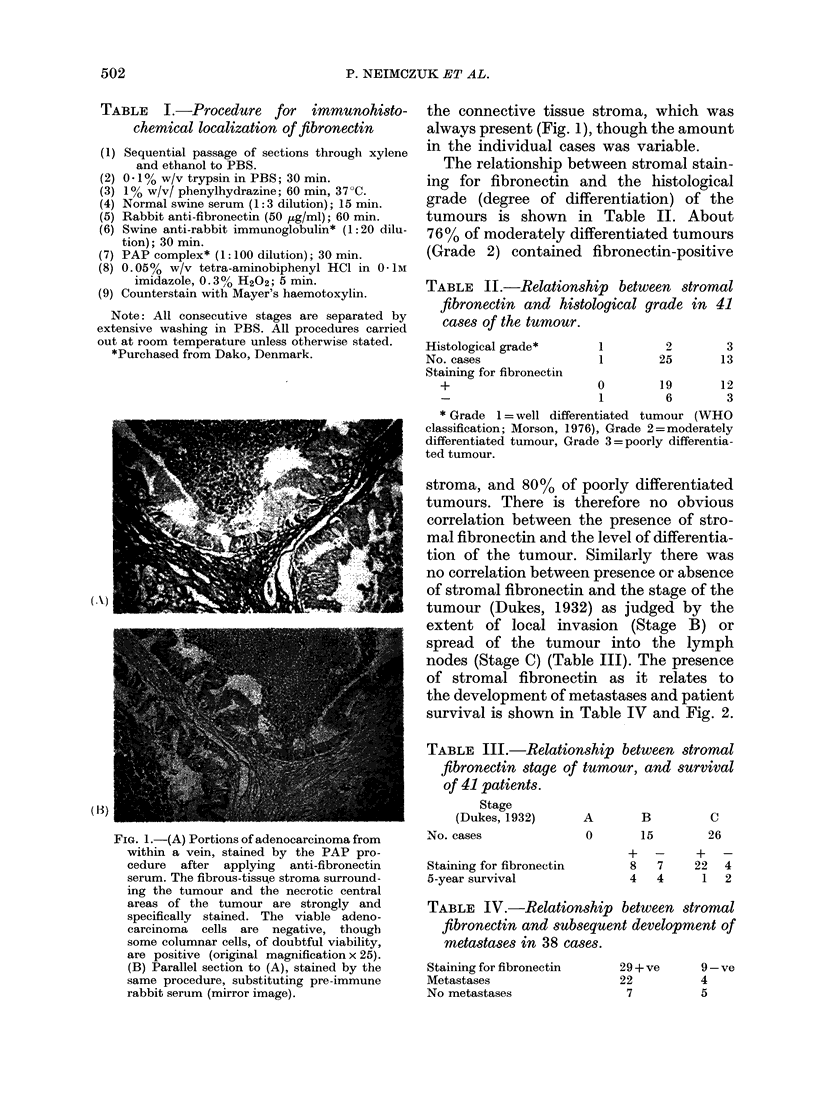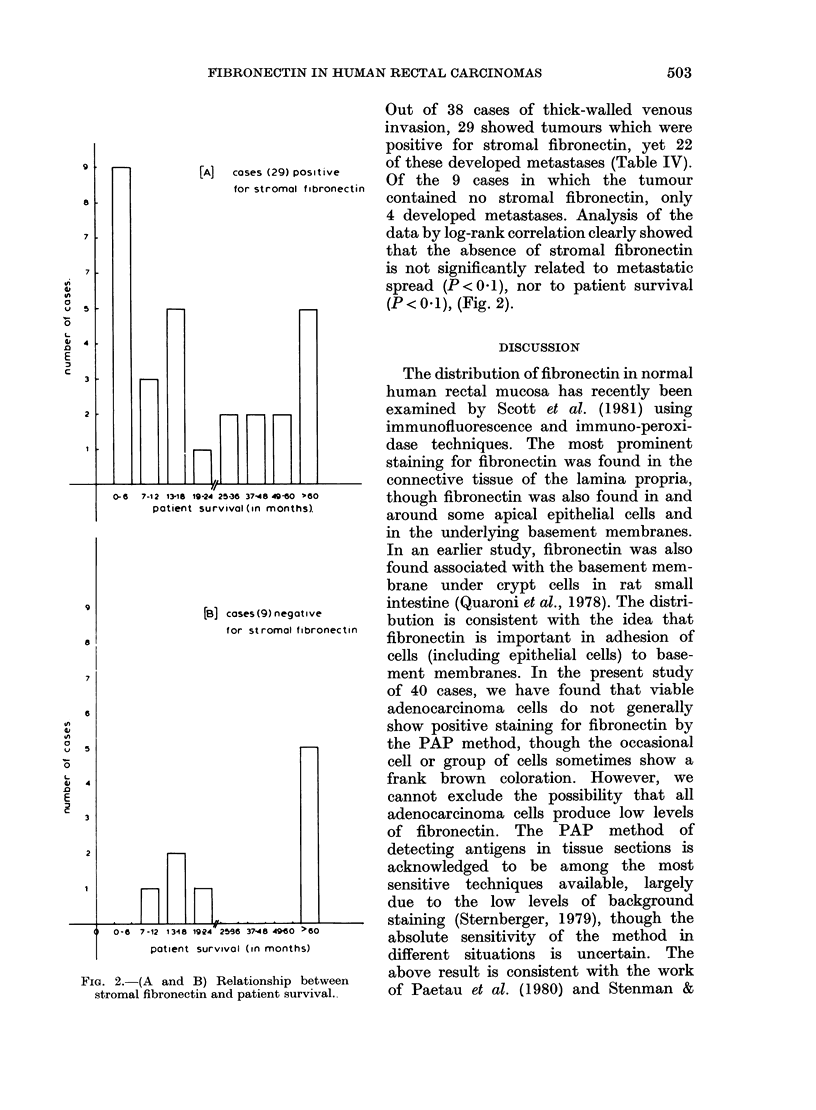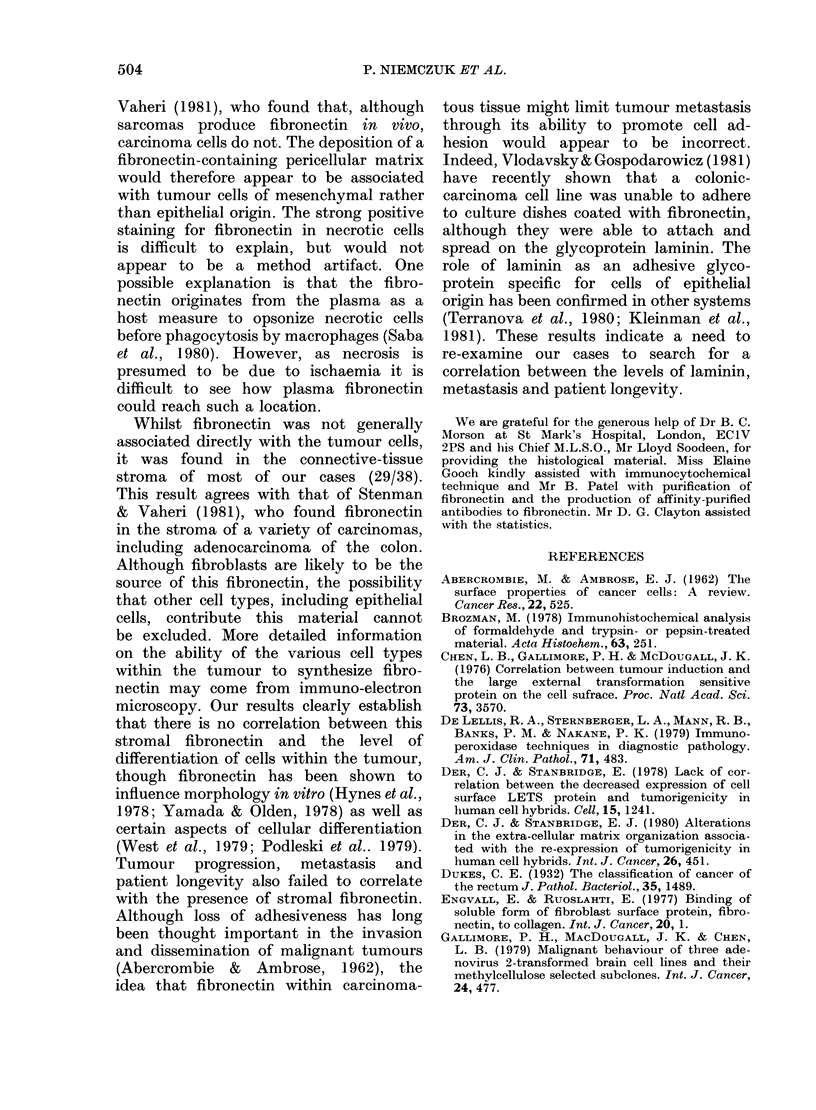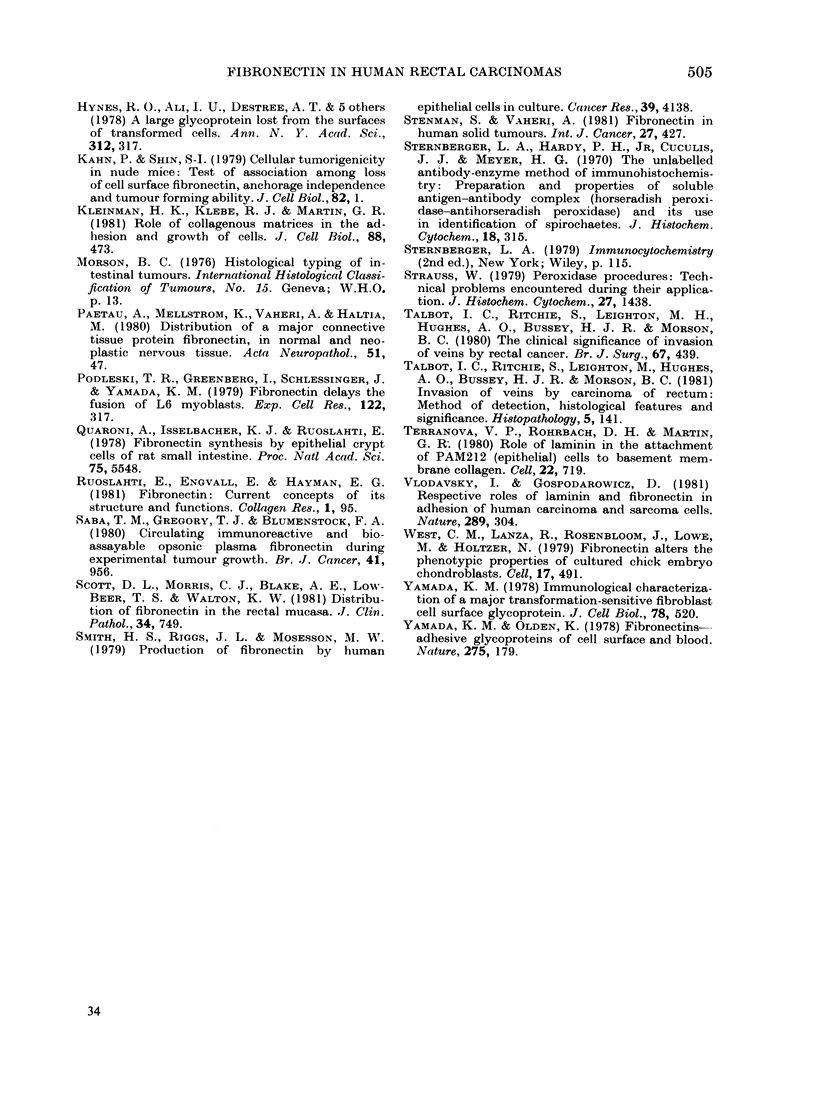# Lack of correlation between metastasis of human rectal carcinoma and the absence of stromal fibronectin.

**DOI:** 10.1038/bjc.1982.85

**Published:** 1982-04

**Authors:** P. Niemczuk, R. M. Perkins, I. C. Talbot, D. R. Critchley

## Abstract

**Images:**


*Br. J. Cancer* (1982) **45**, 500

### LACK OF CORRELATION BETWEEN METASTASIS OF HUMAN RECTAL CARCINOMA AND THE ABSENCE OF STROMAL FIBRONECTIN

P. NIEMCZUK*, R. M. PERKINS*, I. C. TALBOT† AND D. R. CRITCHLEY*

*From the *Department of Biochemistry and the †Department of Pathology, University of Leicester, Leicester LE1 7RH*

Received 26 October 1981 Accepted 15 December 1981

**Summary.—In a retrospective study we have used an immunoperoxidase procedure to localize the glycoprotein fibronectin in human rectal carcinomas, concentrating on tumour invading thick-walled extramural veins. Fibronectin was present in 29 out of 38 cases, in connective tissue stroma, and was not in direct association with the tumour cells, except in areas of necrosis. We found no correlation between the presence or absence of stromal fibronectin and (1) the degree of cellular differentiation within the tumour, (2) tumour progression (Dukes' classification) (3) the subsequent development of metastases and (4) patient longevity. Our results do not support the conclusions from *in vitro* studies (Smith *et al.*, 1979) that the metastatic potential of carcinomas may be partly determined by the ability of tumour cells to synthesize pericellular fibronectin.**

CURRENT INTEREST in the high-mol.-wt glycoprotein fibronectin stems from the observation that, whilst it is associated with the surface of a variety of normal cell lines in culture, particularly those of mesenchymal origin, it is largely absent from their tumour-virus-transformed derivatives (Hynes *et al.*, 1978; Yamada & Olden, 1978). The relationship between loss of surface fibronectin and other characteristics of the transformed phenotype (*e.g.* rounded morphology, loss of contact inhibition) is now clear, and relates to the role of fibronectin as a matrix component important for cell adhesion (Ruoslahti *et al.*, 1981; Kleinman *et al.*, 1981). However, the correlation, if any, between loss of fibronectin *in vitro* and increased tumorigenic potential of transformed cells *in vivo* has been the subject of conflicting reports (Chen *et al.*, 1976; Gallimore *et al.*, 1979; Der & Stanbridge 1978, 1980; Kahn & Shin 1979). In a recent study, Smith *et al.* (1979) found that human epithelial cell lines derived from primary carcinomas or non-malignant tissues showed extensive deposition of matrix fibronectin, whereas lines from metastatic carcinomas expressed little such material. One interpretation of these findings is that a better correlation exists between loss of fibronectin and increased metastatic potential than with tumorigenicity *per se*. However, the need to examine human tumour material directly and avoid possible artifacts generated by taking cells into culture is obvious.

In a previous study of carcinoma of rectum, Talbot *et al.* (1980) showed that primary adenocarcinoma was invading rectal veins in 52% of 703 cases. The prognosis was found to depend on the extent of venous invasion, and was particularly poor when there was spread of tumour into large (thick-walled) extramural veins (Talbot *et al.*, 1981). However, 15/91 patients with this type of extensive venous invasion did in fact survive for

Correspondence to: Dr D. R. Critchley, Dept. of Biochemistry, University of Leicester, Leicester LE1 7RH.

5 years, indicating that spread of the primary tumour into veins cannot be regarded as an invariably sinister event. Whether this is because of variation in the intrinsic nature of the invading adenocarcinoma or due to variation in reactivity of the host tissues at the primary or potential secondary sites is unclear. One possibility is that the adhesiveness and cohesion of the adenocarcinoma cells within invaded veins is greater in some tumours than in others. Because of the suggestions from *in vitro* evidence that cell adhesion is mediated by fibronectin we decided to examine tissue from the cases of rectal adenocarcinoma previously investigated by Talbot *et al.* (1980, 1981) to determine whether any correlation exists between the absence of fibronectin from tumour within extramural veins, metastatic spread and patient longevity.

### MATERIALS AND METHODS

*Tissue sections.*—Five-$\mu$m paraffin sections were cut from blocks of the same cases of surgically excised rectal adenocarcinoma that were previously investigated by Talbot *et al.* (1980, 1981). Only those blocks which included invasion of thick-walled extramural veins by the tumour were studied. (The material is held at the Department of Pathology, St Mark's Hospital, London.)

*Anti-serum to human plasma fibronectin.*—Fibronectin was purified from outdated citrated human plasma by gelatin–Sepharose chromatography (Engvall & Ruoslahti, 1977) followed by preparative SDS-polyacrylamide-gel electrophoresis. The protein recovered by electroelution gave a single band on 7% SDS-polyacrylamide gels. Rabbits were injected s.c. with 1 mg of fibronectin in complete Freund's adjuvant, followed by 2 booster injections (0·5 mg each) at 2-week intervals. The animals were bled 10 days later, and the antiserum characterized by immunodiffusion and immunoelectrophoresis. Whilst the antiserum cross-reacted with both urea-eluted and arginine-eluted fibronectin, as well as with human plasma, it did not cross-react with human serum albumin, fibrinogen, fibronectin-depleted plasma or gelatin. Anti-fibronectin antibodies were subsequently purified by affinity chromatography on a fibronectin–Sepharose column, as described by Yamada (1978).

*Immunohistochemical localization of fibronectin.*—Sections of biopsy material were stained for fibronectin using a 3-layered-bridge technique with a peroxidase anti-peroxidase (PAP) complex (Sternberger *et al.*, 1970). Some of the improvements suggested by Strauss (1979) were used, including the use of phenylhydrazine to block endogenous peroxidase activity, and 0·1M imidazole buffer (pH 7·0) to improve the staining with 3,4,3′4′, tetra-aminobiphenyl hydrochloride. In addition, normal serum from the species supplying the secondary antibody (swine) was used to block nonspecific tissue-binding sites as suggested by De Lellis *et al.* (1979). The swine serum was first depleted of fibronectin by passage through two gelatin–Sepharose columns. As the tissues had been fixed in formalin, all sections were treated with trypsin to unmask potential antigenic sites (Brozman, 1978), though pilot experiments showed that this step did not affect the intensity of the staining for fibronectin. Staining was optimal with an anti-fibronectin concentration of 50 $\mu$g/ml, and was quantitatively removed by adsorbing the antibody with fibronectin. There was no staining in the presence of non-immune immunoglobulins (50 $\mu$g/ml). The final staining protocol adopted is given in Table I. Sections from each case were stained both with anti-fibronectin and non-immune immunoglobulins to determine the presence or absence of fibronectin from tumour within thick-walled extramural veins.

### RESULTS

Staining of tumour in thick-walled extramural veins for fibronectin showed the following characteristics. Clearly viable tumour cells were generally substantially unstained for fibronectin, though occasional cells showed definite positive staining. There was no evidence that tumour cells were associated with a pericellular matrix of fibronectin (Fig. 1). In contrast, necrotic cells, when present, gave strong positive staining for fibronectin, whilst the corresponding control sections were uniformly negative. The bulk of the fibronectin was generally associated with

TABLE I.—*Procedure for immunohistochemical localization of fibronectin*

(1) Sequential passage of sections through xylene and ethanol to PBS.
(2) 0·1% w/v trypsin in PBS; 30 min.
(3) 1% w/v/ phenylhydrazine; 60 min, 37°C.
(4) Normal swine serum (1:3 dilution); 15 min.
(5) Rabbit anti-fibronectin (50 μg/ml); 60 min.
(6) Swine anti-rabbit immunoglobulin* (1:20 dilution); 30 min.
(7) PAP complex* (1:100 dilution); 30 min.
(8) 0.05% w/v tetra-aminobiphenyl HCl in 0·1M imidazole, 0.3% $H_2O_2$; 5 min.
(9) Counterstain with Mayer's haemotoxylin.

Note: All consecutive stages are separated by extensive washing in PBS. All procedures carried out at room temperature unless otherwise stated.
*Purchased from Dako, Denmark.

(A)

(B)

FIG. 1.—(A) Portions of adenocarcinoma from within a vein, stained by the PAP procedure after applying anti-fibronectin serum. The fibrous-tissue stroma surrounding the tumour and the necrotic central areas of the tumour are strongly and specifically stained. The viable adenocarcinoma cells are negative, though some columnar cells, of doubtful viability, are positive (original magnification × 25). (B) Parallel section to (A), stained by the same procedure, substituting pre-immune rabbit serum (mirror image).

the connective tissue stroma, which was always present (Fig. 1), though the amount in the individual cases was variable.

The relationship between stromal staining for fibronectin and the histological grade (degree of differentiation) of the tumours is shown in Table II. About 76% of moderately differentiated tumours (Grade 2) contained fibronectin-positive stroma, and 80% of poorly differentiated tumours. There is therefore no obvious correlation between the presence of stromal fibronectin and the level of differentiation of the tumour. Similarly there was no correlation between presence or absence of stromal fibronectin and the stage of the tumour (Dukes, 1932) as judged by the extent of local invasion (Stage B) or spread of the tumour into the lymph nodes (Stage C) (Table III). The presence of stromal fibronectin as it relates to the development of metastases and patient survival is shown in Table IV and Fig. 2.

TABLE II.—*Relationship between stromal fibronectin and histological grade in 41 cases of the tumour.*

| | | | |
|---|---|---|---|
| Histological grade* | 1 | 2 | 3 |
| No. cases | 1 | 25 | 13 |
| Staining for fibronectin | | | |
| + | 0 | 19 | 12 |
| − | 1 | 6 | 3 |

* Grade 1 = well differentiated tumour (WHO classification; Morson, 1976), Grade 2 = moderately differentiated tumour, Grade 3 = poorly differentiated tumour.

TABLE III.—*Relationship between stromal fibronectin stage of tumour, and survival of 41 patients.*

| Stage (Dukes, 1932) | A | B | | C | |
|---|---|---|---|---|---|
| No. cases | 0 | 15 | | 26 | |
| | | + | − | + | − |
| Staining for fibronectin | | 8 | 7 | 22 | 4 |
| 5-year survival | | 4 | 4 | 1 | 2 |

TABLE IV.—*Relationship between stromal fibronectin and subsequent development of metastases in 38 cases.*

| | | |
|---|---|---|
| Staining for fibronectin | 29 + ve | 9 − ve |
| Metastases | 22 | 4 |
| No metastases | 7 | 5 |

FIG. 2.—(A and B) Relationship between stromal fibronectin and patient survival..

Out of 38 cases of thick-walled venous invasion, 29 showed tumours which were positive for stromal fibronectin, yet 22 of these developed metastases (Table IV). Of the 9 cases in which the tumour contained no stromal fibronectin, only 4 developed metastases. Analysis of the data by log-rank correlation clearly showed that the absence of stromal fibronectin is not significantly related to metastatic spread ($P < 0{\cdot}1$), nor to patient survival ($P < 0{\cdot}1$), (Fig. 2).

### DISCUSSION

The distribution of fibronectin in normal human rectal mucosa has recently been examined by Scott *et al.* (1981) using immunofluorescence and immuno-peroxidase techniques. The most prominent staining for fibronectin was found in the connective tissue of the lamina propria, though fibronectin was also found in and around some apical epithelial cells and in the underlying basement membranes. In an earlier study, fibronectin was also found associated with the basement membrane under crypt cells in rat small intestine (Quaroni *et al.*, 1978). The distribution is consistent with the idea that fibronectin is important in adhesion of cells (including epithelial cells) to basement membranes. In the present study of 40 cases, we have found that viable adenocarcinoma cells do not generally show positive staining for fibronectin by the PAP method, though the occasional cell or group of cells sometimes show a frank brown coloration. However, we cannot exclude the possibility that all adenocarcinoma cells produce low levels of fibronectin. The PAP method of detecting antigens in tissue sections is acknowledged to be among the most sensitive techniques available, largely due to the low levels of background staining (Sternberger, 1979), though the absolute sensitivity of the method in different situations is uncertain. The above result is consistent with the work of Paetau *et al.* (1980) and Stenman &

Vaheri (1981), who found that, although sarcomas produce fibronectin *in vivo*, carcinoma cells do not. The deposition of a fibronectin-containing pericellular matrix would therefore appear to be associated with tumour cells of mesenchymal rather than epithelial origin. The strong positive staining for fibronectin in necrotic cells is difficult to explain, but would not appear to be a method artifact. One possible explanation is that the fibronectin originates from the plasma as a host measure to opsonize necrotic cells before phagocytosis by macrophages (Saba *et al.*, 1980). However, as necrosis is presumed to be due to ischaemia it is difficult to see how plasma fibronectin could reach such a location.

Whilst fibronectin was not generally associated directly with the tumour cells, it was found in the connective-tissue stroma of most of our cases (29/38). This result agrees with that of Stenman & Vaheri (1981), who found fibronectin in the stroma of a variety of carcinomas, including adenocarcinoma of the colon. Although fibroblasts are likely to be the source of this fibronectin, the possibility that other cell types, including epithelial cells, contribute this material cannot be excluded. More detailed information on the ability of the various cell types within the tumour to synthesize fibronectin may come from immuno-electron microscopy. Our results clearly establish that there is no correlation between this stromal fibronectin and the level of differentiation of cells within the tumour, though fibronectin has been shown to influence morphology *in vitro* (Hynes *et al.*, 1978; Yamada & Olden, 1978) as well as certain aspects of cellular differentiation (West *et al.*, 1979; Podleski *et al.*. 1979). Tumour progression, metastasis and patient longevity also failed to correlate with the presence of stromal fibronectin. Although loss of adhesiveness has long been thought important in the invasion and dissemination of malignant tumours (Abercrombie & Ambrose, 1962), the idea that fibronectin within carcinomatous tissue might limit tumour metastasis through its ability to promote cell adhesion would appear to be incorrect. Indeed, Vlodavsky & Gospodarowicz (1981) have recently shown that a colonic-carcinoma cell line was unable to adhere to culture dishes coated with fibronectin, although they were able to attach and spread on the glycoprotein laminin. The role of laminin as an adhesive glycoprotein specific for cells of epithelial origin has been confirmed in other systems (Terranova *et al.*, 1980; Kleinman *et al.*, 1981). These results indicate a need to re-examine our cases to search for a correlation between the levels of laminin, metastasis and patient longevity.

We are grateful for the generous help of Dr B. C. Morson at St Mark's Hospital, London, EC1V 2PS and his Chief M.L.S.O., Mr Lloyd Soodeen, for providing the histological material. Miss Elaine Gooch kindly assisted with immunocytochemical technique and Mr B. Patel with purification of fibronectin and the production of affinity-purified antibodies to fibronectin. Mr D. G. Clayton assisted with the statistics.

### REFERENCES

ABERCROMBIE, M. & AMBROSE, E. J. (1962) The surface properties of cancer cells: A review. *Cancer Res.*, **22**, 525.

BROZMAN, M. (1978) Immunohistochemical analysis of formaldehyde and trypsin- or pepsin-treated material. *Acta Histoehem.*, **63**, 251.

CHEN, L. B., GALLIMORE, P. H. & MCDOUGALL, J. K. (1976) Correlation between tumour induction and the large external transformation sensitive protein on the cell sufrace. *Proc. Natl Acad. Sci.* **73**, 3570.

DE LELLIS, R. A., STERNBERGER, L. A., MANN, R. B., BANKS, P. M. & NAKANE, P. K. (1979) Immunoperoxidase techniques in diagnostic pathology. *Am. J. Clin. Pathol.*, **71**, 483.

DER, C. J. & STANBRIDGE, E. (1978) Lack of correlation between the decreased expression of cell surface LETS protein and tumorigenicity in human cell hybrids. *Cell*, **15**, 1241.

DER, C. J. & STANBRIDGE, E. J. (1980) Alterations in the extra-cellular matrix organization associated with the re-expression of tumorigenicity in human cell hybrids. *Int. J. Cancer*, **26**, 451.

DUKES, C. E. (1932) The classification of cancer of the rectum *J. Pathol. Bacteriol.*, **35**, 1489.

ENGVALL, E. & RUOSLAHTI, E. (1977) Binding of soluble form of fibroblast surface protein, fibronectin, to collagen. *Int. J. Cancer*, **20**, 1.

GALLIMORE, P. H., MACDOUGALL, J. K. & CHEN, L. B. (1979) Malignant behaviour of three adenovirus 2-transformed brain cell lines and their methylcellulose selected subclones. *Int. J. Cancer*, **24**, 477.

HYNES, R. O., ALI, I. U., DESTREE, A. T. & 5 others (1978) A large glycoprotein lost from the surfaces of transformed cells. *Ann. N. Y. Acad. Sci.*, **312**, 317.

KAHN, P. & SHIN, S-I. (1979) Cellular tumorigenicity in nude mice: Test of association among loss of cell surface fibronectin, anchorage independence and tumour forming ability. *J. Cell Biol.*, **82**, 1.

KLEINMAN, H. K., KLEBE, R. J. & MARTIN, G. R. (1981) Role of collagenous matrices in the adhesion and growth of cells. *J. Cell Biol.*, **88**, 473.

MORSON, B. C. (1976) Histological typing of intestinal tumours. *International Histological Classification of Tumours, No. 15.* Geneva; W.H.O. p. 13.

PAETAU, A., MELLSTROM, K., VAHERI, A. & HALTIA, M. (1980) Distribution of a major connective tissue protein fibronectin, in normal and neoplastic nervous tissue. *Acta Neuropathol.*, **51**, 47.

PODLESKI, T. R., GREENBERG, I., SCHLESSINGER, J. & YAMADA, K. M. (1979) Fibronectin delays the fusion of L6 myoblasts. *Exp. Cell Res.*, **122**, 317.

QUARONI, A., ISSELBACHER, K. J. & RUOSLAHTI, E. (1978) Fibronectin synthesis by epithelial crypt cells of rat small intestine. *Proc. Natl Acad. Sci.* **75**, 5548.

RUOSLAHTI, E., ENGVALL, E. & HAYMAN, E. G. (1981) Fibronectin: Current concepts of its structure and functions. *Collagen Res.*, **1**, 95.

SABA, T. M., GREGORY, T. J. & BLUMENSTOCK, F. A. (1980) Circulating immunoreactive and bioassayable opsonic plasma fibronectin during experimental tumour growth. *Br. J. Cancer*, **41**, 956.

SCOTT, D. L., MORRIS, C. J., BLAKE, A. E., LOW-BEER, T. S. & WALTON, K. W. (1981) Distribution of fibronectin in the rectal mucasa. *J. Clin. Pathol.*, **34**, 749.

SMITH, H. S., RIGGS, J. L. & MOSESSON, M. W. (1979) Production of fibronectin by human epithelial cells in culture. *Cancer Res.*, **39**, 4138.

STENMAN, S. & VAHERI, A. (1981) Fibronectin in human solid tumours. *Int. J. Cancer*, **27**, 427.

STERNBERGER, L. A., HARDY, P. H., JR, CUCULIS, J. J. & MEYER, H. G. (1970) The unlabelled antibody-enzyme method of immunohistochemistry: Preparation and properties of soluble antigen–antibody complex (horseradish peroxidase–antihorseradish peroxidase) and its use in identification of spirochaetes. *J. Histochem. Cytochem.*, **18**, 315.

STERNBERGER, L. A. (1979) *Immunocytochemistry* (2nd ed.), New York; Wiley, p. 115.

STRAUSS, W. (1979) Peroxidase procedures: Technical problems encountered during their application. *J. Histochem. Cytochem.*, **27**, 1438.

TALBOT, I. C., RITCHIE, S., LEIGHTON, M. H., HUGHES, A. O., BUSSEY, H. J. R. & MORSON, B. C. (1980) The clinical significance of invasion of veins by rectal cancer. *Br. J. Surg.*, **67**, 439.

TALBOT, I. C., RITCHIE, S., LEIGHTON, M., HUGHES, A. O., BUSSEY, H. J. R. & MORSON, B. C. (1981) Invasion of veins by carcinoma of rectum: Method of detection, histological features and significance. *Histopathology*, **5**, 141.

TERRANOVA, V. P., ROHRBACH, D. H. & MARTIN, G. R. (1980) Role of laminin in the attachment of PAM212 (epithelial) cells to basement membrane collagen. *Cell*, **22**, 719.

VLODAVSKY, I. & GOSPODAROWICZ, D. (1981) Respective roles of laminin and fibronectin in adhesion of human carcinoma and sarcoma cells. *Nature*, **289**, 304.

WEST, C. M., LANZA, R., ROSENBLOOM, J., LOWE, M. & HOLTZER, N. (1979) Fibronectin alters the phenotypic properties of cultured chick embryo chondroblasts. *Cell*, **17**, 491.

YAMADA, K. M. (1978) Immunological characterization of a major transformation-sensitive fibroblast cell surface glycoprotein. *J. Cell Biol.*, **78**, 520.

YAMADA, K. M. & OLDEN, K. (1978) Fibronectins—adhesive glycoproteins of cell surface and blood. *Nature*, **275**, 179.